# Overexpression of mutant EGFR protein indicates a better survival benefit from EGFR-TKI therapy in non-small cell lung cancer

**DOI:** 10.18632/oncotarget.10594

**Published:** 2016-07-13

**Authors:** Yun Ling, Xin Yang, Wenbin Li, Zhuo Li, Lin Yang, Tian Qiu, Lei Guo, Lin Dong, Lin Li, Jianming Ying, Dongmei Lin

**Affiliations:** ^1^ Department of Pathology, National Cancer Center/Cancer Hospital, Chinese Academy of Medical Sciences and Peking Union Medical College, Beijing, China; ^2^ Key laboratory of Carcinogenesis and Translational Research (Ministry of Education), Department of Pathology, Peking University Cancer Hospital & Institute, Beijing, China

**Keywords:** activting EGFR mutation, mutation-specific antibodies, immunohistochemistry, survival, non-small cell lung cancer

## Abstract

**Background:**

Epidermal growth factor receptor (EGFR) is a novel target for therapy in a subset of non-small cell lung cancer (NSCLC). Tumors with *EGFR* mutations showed good response to EGFR tyrosine kinase inhibitors (TKIs). We aimed to identify the discriminating capacity of immunohistochemistry (IHC) to detect EGFR L858R and del E746-A750 mutations in NSCLC patients and predict EGFR TKIs response.

**Methods:**

We collected specimens from 200 patients with NSCLC whose *EGFR* mutation status had been validated by direct DNA sequencing. IHC analyses using EGFR mutation-specific antibodies were employed for all samples. After staining and scoring, the sensitivity, specificity, positive predictive value (PPV) and negative predictive value (NPV) were calculated.

**Results:**

The sensitivity, specificity, PPV, and NPV of IHC using EGFR del E746-A750 and L858R mutation antibodies were 95.0%/95.1%, 85.7%/94.1%, 74.0%/91.8%, and 97.6%/96.5%, respectively. When score 2+ and 3+ were considered as positive, the sensitivity, specificity, PPV, and NPV were 53.3%/36.6%, 99.3%/100%, 97.0%/100%, and 83.2%/65.3%, respectively. The median progression-free survival (PFS) after the start of gefitinib treatment was significantly longer in patients with a high score for mutant EGFR expression than in those with a low score (31.0 versus 13.0 months, p <0.05).

**Conclusions:**

IHC with EGFR mutation-specific antibodies is a promising screening method for detecting *EGFR* mutations in NSCLC patients. Otherwise, quantitative analysis of mutant EGFR expression might also predict the efficacy of TKIs treatment for NSCLC patients harboring sensitive *EGFR* mutation.

## INTRODUCTION

Advanced non–small cell lung cancer (NSCLC) patients carrying activating epidermal growth factor receptor (*EGFR*) mutations markedly respond to EGFR tyrosine kinase inhibitors (TKIs) [1-3]. Sensitizing *EGFR* mutations affect 30%-64% of Asian NSCLC patients, mostly in adenocarcinomas [4, 5]. In-frame deletions in exon 19 and arginine substituting leucine 858 (L858R) in exon 21 are two of the most common *EGFR* mutation types, accounting for about 50% and 44% of *EGFR* mutations. The majority of exon 19 del is del E746-A750) [6, 7, 23].

Molecular methods to detect *EGFR* mutations in formalin fixed tissue specimens include real-time PCR and direct sequencing, whose costs and technical requirements are prohibitive for routine use in most settings. Meanwhile, immunohistochemistry (IHC) staining represents a method already in use by pathologists; relatively low cost and efficiency allow this tool to be used to screen patients routinely. Antibodies targeting mutated EGFR by IHC would enable facile pre-assessments complementing the current molecular tests in NSCLC patients. Two monoclonal antibodies (mAbs) targeting mutated EGFR proteins (E746-A750 deletion in exon 19 and L858R point mutation in exon 21) had been developed and used for immunohistochemical staining [8].

Here, we employed these EGFR mutation-specific monoclonal antibodies to assess *EGFR* mutations in 200 NSCLC specimens, comparing the data with findings revealed by other molecular techniques. Finally, we evaluated the association of EGFR expression levels with efficacy of EGFR-TKIs treatment.

## RESULTS

### Patients characteristics

Of the 200 NSCLC patients, 184 individuals (92.0%) were diagnosed as adenocarcinoma, 9 (4.5%) as squamous cell carcinoma (SCC), 4 (2.0%) as adenosquamous carcinoma and 3 (1.5%) as other types. A median patient age of 58 years was obtained, ranging between 35 and 79 years. The male to female ratio was 1:1. One hundred and ninety samples were obtained by resection and the remaining 10 by biopsy. There were 21 tumors with high differentiation, 94 with moderate differentiation, and 81 with low differentiation. Four biopsy cases had distinguished degree of differentiation because of low percentage of tumor cells (Table 1).

**Table 1:** Clinicopathological features of the patients analyzed for EGFR mutations by IHC assay

Characteristic	Pations(N=200)	
	No.	%
Age(average)	58.2	
Gender		
Male	100	50.0
Female	100	50.0
Types of samples		
Resection	190	95.0
Biopsy	10	5.0
Histology		
Adenocarcinoma	184	92.0
Squamous cell carcinoma	9	4.5
adenosquamous carcinoma	4	2.0
Others	3	1.5
Differentiation		
High	21	10.5
Moderate	94	47.0
poor	81	40.5
Unclassified	4	2.0

### *EGFR* mutations and IHC analysis

**Figure 1:**
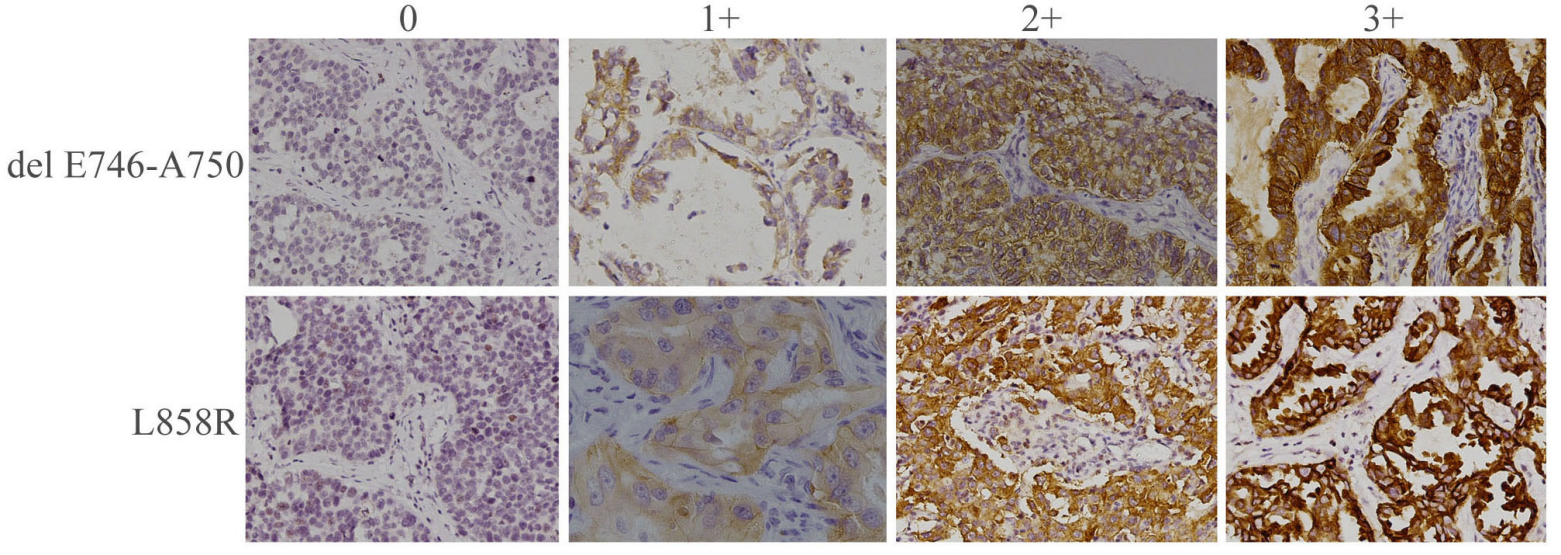
Immunohistochemical staining of human NSCLC tumor samples with antibodies specific for delE746-A750 or L858R mutant forms of EGFR Representative staining patterns for each of the four intensity levels are shown (original magnification,×200).

The two specific antibodies displayed recognizably different immunoreactivities as shown in Figure 1. Mutations detected by EGFR IHC and sequencing are summarized in Table 2. Sequencing analysis detected 60 exon 19 (del E746-A750) deletions, 30 other exon 19 deletions, 82 exon 21 (L858R) mutations and 28 cases without *EGFR* mutation. Of the del E746-A750 deletions detected by sequencing, 57 cases were detected by exon 19 antibody with immunohistochemical score of 1+ to 3+. However, there were only 32 cases detected by exon 19 antibody as strongly positive. Of the 30 cases with other exon 19 deletions, 17 had faint staining (1+) and only one moderate staining (2+) was obtained. Of the L858R mutations detected by sequencing, 78 cases were detected by exon 21 antibody with immunohistochemical scores of 1+ to 3+. However, there were only 32 cases detected by exon 21 antibody with strongly positive.

**Table 2:** Comparison of results of EGFR mutation-specific antibodies and DNA direct sequencing

IHC	L858R		delE746-A750		total
	Seq+	Seq-	Seq+	Seq-	
1+/2+/3+	78	7	57	20	
0	4	111	3	120	
2+/3+	30	0	32	1	
0/1+	52	118	28	139	
Total	82	118	60	140	200

### Specificity and sensitivity of EGFR immunohistochemistry

Sensitivity and specificity of exon 19 antibody were 95.0% and 85.7%, respectively, when positive cases were designated as immunohistochemical scores of 1+ to 3+. Meanwhile, sensitivity and specificity of exon 19 antibody were 53.3% and 99.3%, respectively, when positive cases were designated as immunohistochemical scores of 2+ to 3+. Positive (PPV) and negative (NPV) predictive values were 97.0% and 83.2%, respectively. Excluding the 30 cases with other exon 19 mutations, specificity of exon 19 antibody was 97.3% (107/110).

Sensitivity and specificity of exon 21 antibody were 95.1% and 94.1%, respectively, with positive cases considered for immunohistochemical scores of 1+ to 3+. Meanwhile, sensitivity of exon 21 antibody was 36.6% for a specificity of 100%, when positive cases were designated as immunohistochemical scores of 2+ to 3+. PPV and NPV of 100% and 65.3% were obtained in this case (Table 3).

**Table 3:** The detection accuracy for the EGFR mutation-specific antibodies

EGFR mutation type	Sensitivity (%)	Specificity (%)	PPV(%)	NPV(%)
L858R mutation				
1+/2+/3+	95.1	94.1	91.8	96.5
2+/3+	36.6	100.0	100.0	65.3
Del mutation				
1+/2+/3+	95.0	85.7	74.0	97.6
2+/3+	53.3	99.3	97.0	83.2

PPV: positive predictive value; NPV: negative predictive value.

### Association between EGFR immunostaining data and survival

**Figure 2:**
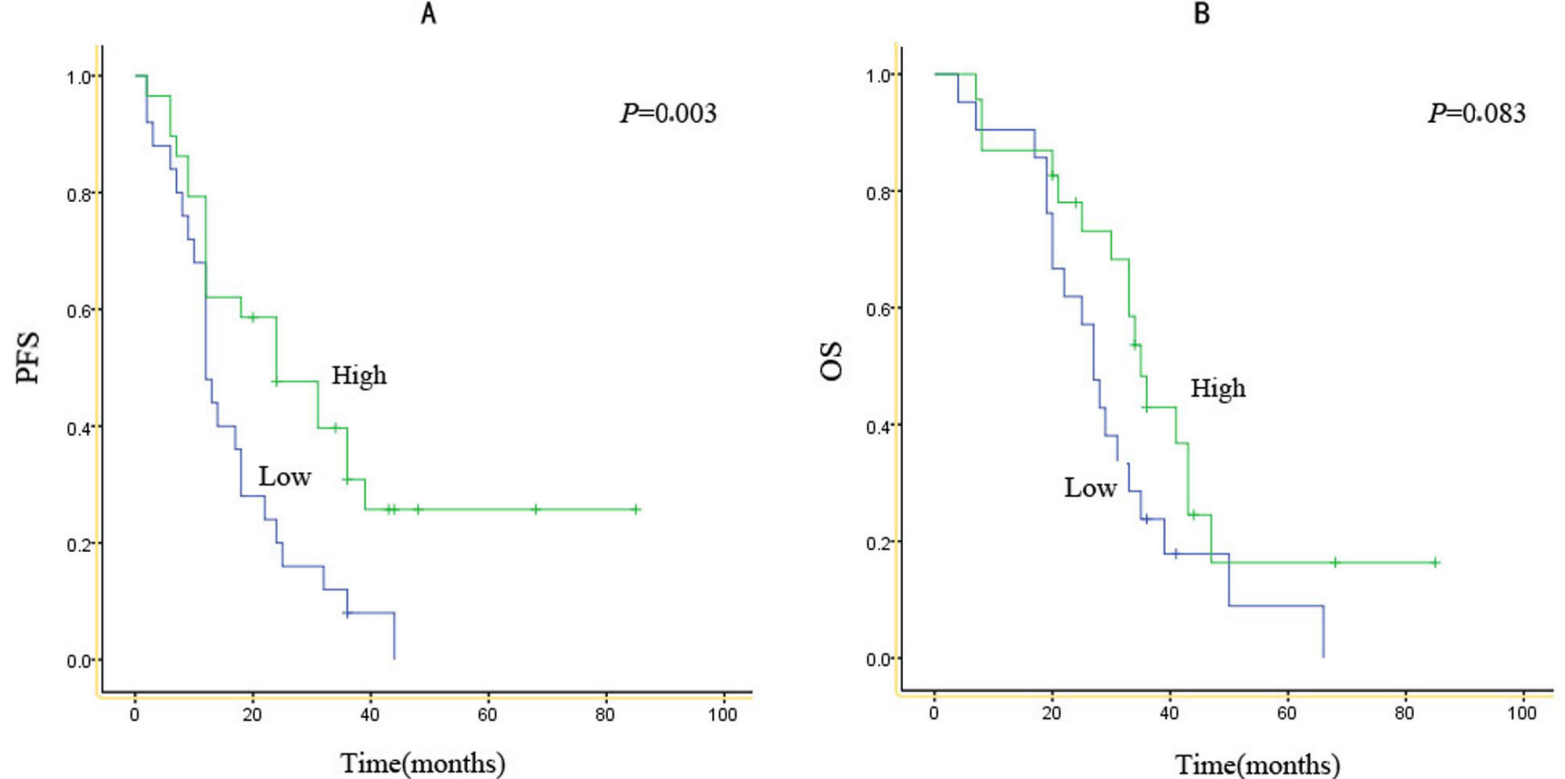
Kaplan-Meier survival curves according to expression score for EGFR mutants.PFS (A) and OS (B) for patients with high or low expression scores for either type of EGFR mutant

In this study, 54 and 44 patients had PFS and OS data, respectively. Follow up averagely lasted 25.7 months, ranging between 11.0 and 85.0 months. Median PFS and OS of 18.0 (2.0–85.0) months and 30.5 (4.0–85.0) months were obtained, respectively. At the end of cut-off data, 10 cases continued to be administered gefitinib. Median durations of gefitinib administration in cases showing high and low scores were 31.0 (2.0–85.0) and 13.0 (2.0–44.0) months, respectively. PFS and OS curves from initial gefitinib treatment are displayed in Figure 2. Gefitinib yielded markedly longer PFS in cases showing high IHC scores for EGFR mutants compared with the values obtained for individuals presenting low scores (31.0 versus 13.0 months, *p*=0.003; Figure 2A); meanwhile, no overt difference in OS was observed (35.0 vs. 27.0 months, *p*=0.083; Figure 2B). Interestingly, high mutant EGFR level (*p*<0.05) was significantly correlated with PFS, as shown by univariate analyses. No other factor assessed showed a significant association with PFS or OS (Table 4). In addition, mutant EGFR score was correlated with PFS with statistical significance (hazard ratio, 2.798; 95% confidence interval, 1.428–5.484; *p*<0.05) independently of age, gender and differentiation (Table 5). However, after accounting for multiple comparisons, associations with OS were not statistically significant for these parameters.

**Table 4:** Factors associated with PFS

Factor	n	Median PFS(m)	p*
Age(yr)			0.854
High(≥60)	28	22.0	
Low(<60)	26	21.9	
Sex			0.969
Male	23	22.1	
Female	31	21.8	
Smoking status			0.669
Former/current	13	13.0	
Never	41	20.0	
Stage			0.462
I	11	25.0	
II	12	24.0	
III	20	14.0	
IV	10	12.0	
Differentiation			0.649
High	2	16.5	
Moderate	31	20.4	
poor	21	24.7	
EGFR-mutant expression score			
High(2+ and 3+)	27	31.0	0.003
Low (0 and 1+)	27	13.0	

* Univariate analysis by log-rank test.

**Table 5:** Multivariate analysis of PFS and OS

	Parameter	HR(95% CI)	P*
PFS	EGFR-mutant expression score(high vs. low)	2.798(1.428-5.484)	<0.05
	Age	1.052(0.564-1.963)	0.873
	Gender Differentiation	0.853(0.442-1.647)	0.636
	High vs. moderate	2.110(0.448-9.950)	0.345
	Moderate vs. poor	0.800(0.405-1.579)	0.520
OS	EGFR-mutant expression score(high vs. low)	1.794(0.887-3.626)	0.104
	Age	1.198(0.582-2.466)	0.625
	Gender	1.547(0.730-3.281)	0.255
	Differentiation		
	High vs. moderate	1.647(0.183-14.759)	0.658
	Moderate vs. poor	0.818(0.404-1.656)	0.576

* Multivariate analysis by Cox proportional hazards model.

## DISCUSSION

Accurate and rapid methods to determine the EGFR status are needed for lung carcinoma patients. Here, sensitivity and specificity of two EGFR mutation specific antibodies were assessed in comparison to sequencing data in Chinese NSCLC patients. Using an IHC score of ≥ 2+ as a criterion of positivity, each antibody had high specificity for E746-A750 del and L858R (99.3% and 100.0%, respectively) but low sensitivity (36.6% and 53.3%, respectively), and individuals highly expressing EGFR mutants showed better survival benefit from EGFR-TKI therapy.

Antibodies specifically targeting EGFR with deleted E746-A750 and L858R point mutation, respectively, showed 92% sensitivity for NSCLC tissue specimens in IHC [8]. Meanwhile, others found IHC sensitivities and specificities of 47-92% and 96-99%, respectively, for these antibodies in NSCLC specimens [9-14]. Indeed, scarce EGFR mutations, e.g. L747-T751 and L747-A750 in exon 19, are weakly or not reactive to the latter antibody molecules. Here, IHC and DNA-based tests on lung cancer samples were utilized; as shown above, sensitivity and specificity of 95.1% and 94.1% were obtained for anti-L858R antibodies, respectively; these values were 95.0% and 85.7%, respectively for anti-E746-A750 del antibodies. The relatively lower specificity of exon 19 del IHC could be explained by that several other deletions present in this sequence may not be targeted by the antibodies used in the current study. Indeed less frequently encountered mutations have been described in EGFR. While Yu et al [8] found only 2 patients harboring rare deletion mutations in exon 19; as shown above, a total 30 specimens were found with rare deletion mutations in exon 19. In these samples, there were 17 cases with faint staining (1+) and one with moderate staining (2+). The specificity reached 97.3% for the E746-A750 del antibody when samples with uncommon deletions in exon 19 were excluded for analysis. Our results showed that high positive immunostaining (2+ and 3+) was markedly associated with DNA-based test data, in agreement with previous reports [14-16], indicating EGFR-TKI treatment using gefitinib could start as soon as possible in individuals with strong positive IHC signals for anti-EGFR mutation antibody molecules, while DNA-based tests should be employed to confirm EGFR status only in individuals with ambiguous IHC data. Indeed, considering IHC screening performances, and the importance to spot all EGFR mutation variants for optimal therapeutic decisions, IHC is unable to replace molecular analyses. However, IHC can serve as first-line or concomitant screening tool in the routine assessment of samples.

Unlike the current DNA sequencing or ARMS approaches, IHC relies on staining strength for single cancer cells in lieu of data acquired for the entire tissue specimen. Therefore, it is easy to miss mutations by DNA sequencing in cancer tissue specimens with low rate of EGFR-mutated cells, which are detectable by IHC. Importantly, small biopsy specimens frequently yield insufficient high quality DNA for tests. Our data showed that one biopsy tissue fragment which had abundant lymphocytes and small amounts of tumor cells showed immunohistochemical signals but was negative in DNA-sequencing and ARMS examinations. Furthermore the currently used DNA sequencing or ARMS approaches cannot display tumor heterogeneity, while IHC provides various intensities and percentages of tumor cells and allows more comprehensive molecular diagnostics. The main advantage of the IHC method is that morphology and staining signals of tumor cells can be observed simultaneously, and tumor heterogeneity can be searched *in situ*, while Sanger sequencing or ARMS methods proceed with a whole lysate and cannot distinguish normal and wild tumor cells from mutant tumor cells. Thus, it is difficult to determine tumor heterogeneity using PCR-based methods with whole tissue to test gene mutations, unlike IHC.

As shown above, immunohistochemical intensity of mutated EGFR was correlated with PFS when gefitinib administration started. The result was similar to other articles [17]. These findings suggest the intensity score for mutated EGFR would be helpful for efficacy prediction of EGFR-TKIs in individuals with NSCLC carrying EGFR mutations. EGFR-TKI efficacy differs in NSCLC individuals with EGFR mutations; however, a molecular marker which can predict therapeutic response remains unknown. Some studies have demonstrated that T790M mutant EGFR and MET amplification are correlated with resistance induced by EGFR-TKIs in NSCLC subjects with mutated EGFR [18-21]. Tumor heterogeneity has been commonly recognized as another reason for discrepant response to EGFR-TKI treatment among EGFR-mutant patients. However, previous comprehension of tumor heterogeneity was more limited to the proportion of cancer cells harboring EGFR-sensitive mutations. However, our previous study demonstrated that EGFR amplification might be the fundamental cause for varied EGFR-TKI response rather than the cancer cell proportion [22]. PFS improvement after gefitinib administration to individuals with high expression scores for mutated EGFR was not reflected by OS data. It is presumed that the limited sample size could be one of the reasons why a significant difference in OS was not detected.

Overall, mutation-specific IHC for E746_A750 deletion and L858R point mutations in EGFR is highly reliable, with the promise to constitute a rapid screening assay for mutations in individuals with NSCLC, also predicting gefitinib therapeutic efficacy for EGFR mutant NSCLC.

## MATERIALS AND METHODS

### Patients

Two hundred subjects with available EGFR molecular data were assessed, including 190 surgical resections and 10 biopsies from Jan 2003 to Dec 2010. All cases were formalin-fixed and paraffin-embedded (FFPE) specimens. Most cases classified as adenocarcinoma, mixed subtype, as determined by pathological evaluation by an expert. Sections with less than half tumor cells were labeled, further tumor macro-dissection was carried out for cancer cell population enrichment prior to molecular assays. A total of 190 resection samples were selected for tissue microarray (TMA) construction. Approval for this research was obtained from the Institutional Review Board of Cancer Hospital, Chinese Academy of Medical Sciences. All participants provided signed informed consent.

### DNA extraction and mutation analysis

*EGFR* mutation testing was conducted as previously described [9]. Briefly, macro-dissection was performed to obtain tissue samples containing more than half of cancer cells. Genomic DNA was obtained with the QIAamp DNA Mini Tissue kit (Qiagen, Germany) according to the manufacturer's instructions. Exons 19 and 21 encoding the tyrosine kinase domain of the *EGFR* gene were identified by direct DNA sequencing. Primers for exon 19 were 5'-CATGTGGCACCATCTCACA-3' (forward primer) and 5'-CAGCTGCCAGACATGAGAA-3' (reverse primer); those of exon 21 were 5'-CCTCACAGCAGGGTCTTCTC-3' (forward primer) and 5'-TGCCTCCTTCTGC ATGGTA-3' (reverse primer). PCR was carried out in 25 μL PCR reactions with 200 ng template DNA and annealing at 72°C for 35 cycles. DNA sequencing was carried out using ABI 3500xl Genetic Analyzer (Applied Biosystems, Foster City, CA). Deletion of E746-A750 in exon 19 (n=60, 30.0%) and mutation of L858R in exon 21 (n=82, 41.0%) were considered to be positive. Other deletions in exon 19 (n=30, 15%) and wild-type sequences (n=28, 14.0%) were considered to be negative.

### Tissue microarray (TMA) analyses

On the harvested block, two paraffin cores of 2 mm diameter were obtained in every sample, and precisely ranged into fresh recipient TMA blocks with the trephine apparatus (Mitogen, Minicore, France) following the manufacturer's protocols.

### Immunohistochemical analysis

EGFR E746-A750 deletion-specific and L858R mutant-specific (Cell Signaling Technology, Inc.) antibodies were used. IHC staining of TMAs was performed on BenchMark XT (Ventana Medical Systems, USA) using OptiView DAB IHC detection kit. Briefly, after deparaffinization, 4μm-thick sections of TMAs submitted to antigen retrieval were incubated with antibodies at 37^o^C (16 min) after endogenous peroxidase quenching, with nuclei stained with hematoxylin. IHC detection levels were rated as follows: 0, no/faint signals in less than 10% of tumor cells; 1+, 2+, and 3+ were considered for weak, moderate and intense signals in more than 10% of tumor cells, respectively. Scores of 0, 1+, and 2+ to 3+ were deemed to be negative, slightly positive/low expression, and strongly positive/high expression, respectively. IHC data were reviewed by two pathology independent specialists.

### Statistical analysis

The sensitivity and specificity of EGFR test by IHC was determined in comparison with PCR-based results. Statistics were carried out using SPSS software (version 16.0 of SPSS, Chicago, IL, USA).

## Abbreviations

EGFR: Epidermal growth factor receptor
NSCLC: non-small cell lung cancer
TKIs: EGFR tyrosine kinase inhibitors
IHC: immunohistochemical
PPV: positive predictive value
NPV: negative predictive value
PFS: progression-free survival
OS: overall survival
FFPE: formalin-fixed, paraffin embedded tissues
SCC: squamous cell carcinoma
TMA: tissue microarray.
